# Bioinformatics/Medical Informatics in Traditional Medicine and Integrative Medicine

**DOI:** 10.1155/2015/460490

**Published:** 2015-11-04

**Authors:** Zhaohui Liang, Xiangji Huang, Byeongsang Oh, Josiah Poon

**Affiliations:** ^1^York University, Toronto, ON, Canada; ^2^School of Computer Science, Central China Normal University, Wuhan, China; ^3^School of Information Technology, York University, Toronto, ON, Canada; ^4^Sydney Medical School, The University of Sydney, Sydney, Australia; ^5^School of Information Technologies, Faculty of Engineering and Information Technologies, The University of Sydney, Sydney, Australia

Traditional Chinese Medicine (TCM) and integrative medicine are key components of the cultural heritage from Eastern Asia with thousands-of-years history in research and healthcare delivery. Traditional oriental medicine contributes significantly to the prosperity of Chinese and Eastern Asian culture. After the introduction of western biomedicine to Asia, traditional medicine still plays an important role in the healthcare system of many Asian countries and integrated with the mainstream medical treatments as a new track of healthcare named as integrative medicine. With the current trend of globalization, traditional medicine and integrative medicine are receiving gradual acceptance in the Western world. As a result, studies on traditional medicine attract more and more attention from researchers with various knowledge backgrounds and technologies.

Medical informatics is a new interdisciplinary branch in medical science when computer science and information technology are combined with research of health science. The application of medical informatics that has extended to the studies of traditional medicine and other therapies of complementary and alternative medicine (CAM).

The special issue supported by this journal provides a forum for traditional and integrative medical researchers and practitioners to share and exchange their new ideas on using computer science and information technology to explore and solve problems in healthcare. It is proposed with the Fifth International Workshop on Information Technology for Chinese Medicine (ITCM 2014) in Guangzhou, China, on 12 to 14 December 2014. The workshop is in conjunction with the 2014 IEEE International Conference on Bioinformatics and Biomedicine (BIBM'14), which was held in Belfast, UK, on 2 to 5 November 2014. Professor Xusheng Liu, Professor Honglai Zhang, and Professor Guozheng Li cochaired the workshop. The conference invited top experts from the US, UK, Australia, and Hong Kong to present their inspiring research outcomes and prospect the future of traditional and integrative medicine. However, numerous scientists and researchers were unable to introduce their excellent idea due to time limit of the workshop.

The ITCM 2014 received about 100 submissions. All papers were anonymously reviewed by members of the IEEE conference organization committee. The accepted papers were published in the Proceedings of the 2014 IEEE International Conference on Bioinformatics and Biomedicine Workshops (IEEE-BIBMW) (ISBN 978-1-4799-1309-1). Just a few excellent papers were later invited to submit the extension version to the special issue alongside external submissions for consideration of publishing. This special issue has received 37 submissions. All papers have gone through rigorous view, and only 10 of them (27%) are finally accepted for publication.

This special issue reflects the up-to-date progress in applications of information technology to traditional and integrative medicine. The papers are categorized to represent the four aspects of medical informatics research of the discipline. In the paper entitled “Standardization of Syndrome Differentiation Defined by Traditional Chinese Medicine in Operative Breast Cancer: A Modified Delphi Study,” Q. Guo and Q. Chen present their research on TCM syndromes. Five papers are selected to demonstrate the research progress in disease diagnosis and treatment. G.-X. Shi et al. report a clinical study on vascular dementia. Z. Chen presents a new mathematics method to explore the classical theory of five elements in TCM in his work “Researches on Mathematical Relationship of Five Elements of Containing Notes and Fibonacci Sequence Modulo 5.” In “Syndrome Differentiation Analysis on MARS500 Data of Traditional Chinese Medicine,” Y.-Z. Li et al. succeed to use MARS500 to process the data of traditional medicine. The paper entitled “Detecting Disease in Radiographs with Intuitive Confidence” by S. Jaeger introduces the new idea to use informatics method to detect disease. Three papers are about information processing of traditional medicine. The paper entitled “Patterns Exploration on Patterns of Empirical Herbal Formula of Chinese Medicine by Association Rules” by L. Huang et al. used association rules to retrieve patterns from classical traditional medical formula. B. Zhang et al. proposed a bioinformatics approach to explore the latent patterns from conventional formula Shuang-Huang-Lian in their work “Using Bioinformatics Approach to Explore the Pharmacological Mechanisms of Multiple Ingredients in* Shuang-Huang-Lian*.” The paper entitled “Pulse-Diagnosis Signals Analysis of Fatty Liver Disease and Cirrhosis Patients by Using Machine Learning” by N. Wang et al. introduces new data mining method to process diagnostic data of liver disease. Finally, the paper entitled “An Ensemble Learning Based Framework for Traditional Chinese Medicine Data Analysis with ICD-10 Labels” by G. Zhang et al. and the paper entitled “ISMAC: An Intelligent System for Customized Clinical Case Management and Analysis” introduce the applications of machine learning to electronic data analysis of traditional medicine.

